# Investigating the Clinico-Etiological Profile of Pediatric Pancytopenia: A Prospective Study in a Tertiary Care Center

**DOI:** 10.7759/cureus.67875

**Published:** 2024-08-26

**Authors:** Neha Dattatreya Nadig, Priyank Rajan, Venkata Sai Kiran Kolahalam, Shreya Peddi, Tanisha Rathi, Lakshya Choudhary, Mohit Agrawal, Tejaswee Lohakare, Gaurav Mittal

**Affiliations:** 1 General Medicine, Adichunchanagiri Institute of Medical Sciences, B.G Nagara, IND; 2 Paediatrics and Child Health, Bombay Hospital Institute of Medical Sciences, Mumbai, IND; 3 Paediatric Hematology, Oncology and Bone Marrow Transplant, SRCC Childrens Hospital, Mumbai, IND; 4 Immunohematology and Blood Transfusion, B.J. Medical College and Civil Hospital, Ahmedabad, IND; 5 Paediatrics, Prathima Institute of Medical Sciences, Nagunoor, IND; 6 Internal Medicine, Mahatma Gandhi Institute of Medical Sciences, Wardha, IND; 7 Physical Medicine and Rehabilitation, S.S. Agrawal Institute of Physiotherapy and Medical Care Education, Navsari, IND; 8 Child Health Nursing, Smt. Radhikabai Meghe Memorial College of Nursing, Wardha, IND; 9 Research and Development, Students Network Organization, Mumbai, IND

**Keywords:** pediatric age group, bone marrow biopsy, aplastic anemia, etiology, clinical features, pancytopenia

## Abstract

Background

A decrease in red blood cells, white blood cells, and platelets is known as pancytopenia, and it is frequently caused by a variety of illnesses such as bone marrow failure, malnutrition, infections, autoimmune diseases, or blood malignancies. The purpose of this study was to investigate the hematological, clinical, and causative aspects of pancytopenia in children ranging in age from one month to 18 years.

Methodology

At the Department of Pediatrics, a 12-month prospective observational research involving 70 pediatric patients with pancytopenia was carried out. Documentation included demographic information, clinical manifestations, history of vaccinations, and particular disease features. The study evaluated the frequency of several reasons (autoimmune, neoplastic, nutritional, infectious, and others) that lead to pancytopenia.

Results

The mean age of the 70 patients was 5.08 ± 3.22 years, comprising 30 males (42.86%) and 40 females (57.14%). Fever (50 cases, 71.43%), arthralgias (39 cases, 55.71%), weight loss (11 cases, 15.71%), and failure to thrive (13 cases, 18.57%) were among the most common symptoms. Examinations of the bone marrow revealed that 29 patients (41.43%) had aplastic alterations, 20 patients (28.57%) had hyperplastic abnormalities, and 21 patients (30%) had normal cellularity. Fourteen patients (20%) had nutritional reasons, mostly megaloblastic anemia, and six patients (8.57%) suffering from neoplastic disorders had acute lymphoblastic leukemia (ALL). Hemolytic anemia, parvovirus B19, miliary tuberculosis (TB), aplastic anemia, and systemic lupus erythematosus were among the other noteworthy etiologies.

Conclusion

Many cases of pediatric pancytopenia in the region are preventable. Greater awareness and appropriate diagnostic strategies are crucial for the early identification and management of these conditions.

## Introduction

Pancytopenia is a notable clinical and hematological disorder characterized by a decrease in the number of platelets, white blood cells, and red blood cells in peripheral blood [1]. This condition presents with leukopenia (total leukocyte count (TLC) ≤4 × 10^9/L), thrombocytopenia (platelet count ≤150 × 10^9/L), and anemia (hemoglobin (Hb) below age-specific standards). The symptoms typically reflect these cytopenias and include fatigue, pallor, recurrent infections, and bleeding tendencies, all of which can significantly affect a child's health [2].

Pancytopenia in children can result from a wide spectrum of causes, ranging from transient viral infections that temporarily suppress bone marrow function to life-threatening malignancies that infiltrate and replace normal marrow tissue [3]. Common etiologies include aplastic anemia, leukemia, myelodysplastic syndromes, and infections such as HIV, tuberculosis, and Epstein-Barr virus [3,4]. The presentation of bone marrow in pancytopenic children can vary from normocellular with non-specific changes to hypercellular with complete replacement by malignant cells [5]. Therefore, bone marrow aspiration and biopsy are critical components of the diagnostic workup in pancytopenic patients, providing essential insights into the underlying cause [5].

The epidemiology of pancytopenia varies globally, with differences influenced by geographic location, socioeconomic status, and the prevalence of infectious diseases. In developing countries, infections and nutritional deficiencies are more common causes, whereas in developed regions, pancytopenia is more frequently associated with malignancies and inherited bone marrow failure syndromes [3,4]. Timely and accurate diagnosis of the underlying etiology is crucial, as it directly influences treatment strategies and prognosis. Delays in diagnosis and treatment can result in severe complications, including life-threatening infections, profound anemia, and hemorrhage [1].

Despite the clinical significance of pancytopenia, literature focusing specifically on pediatric populations remains limited. While adult studies have provided substantial insights into the clinical and etiological aspects of pancytopenia, there is a notable gap in data concerning children [2]. This study aims to fill this gap by identifying the preventable causes of pancytopenia in children, thereby contributing to better early diagnosis, guiding treatment decisions, and potentially reducing the morbidity and mortality associated with this condition.

## Materials and methods

Study setting and design

This study is a prospective observational study conducted in accordance with STROBE (Strengthening the Reporting of Observational Studies in Epidemiology) guidelines over 12 months in the Department of Pediatrics. The primary objective was to observe and document clinical and hematological parameters in pediatric patients presenting with pancytopenia. The study included all pediatric patients admitted through the outpatient department (OPD) or emergency department who met the study's inclusion criteria.

Selection Criteria

The study's inclusion criteria were children aged one month to 18 years whose blood investigations revealed hemoglobin (Hb) levels below 10 gm/dL, total leukocyte count (TLC) below 4000/mm³, and platelet count below 100,000/mm³. Only patients whose parents or guardians provided informed consent were included. Exclusion criteria included children younger than one month or older than 18 years, those diagnosed with aplastic anemia or leukemia, individuals with recent blood transfusions, patients undergoing chemotherapy or radiotherapy, and those who declined to provide consent.

Data sources and variables

Patients were enrolled in the study upon obtaining informed written consent from their parents or guardians. As this was an observational study, pediatricians performed a thorough history and clinical examination as part of the standard care, focusing on clinical features such as age at diagnosis, medication history, fever, and signs of organomegaly like lymphadenopathy and hepatomegaly. These observations were documented without any alteration in the standard diagnostic and treatment protocols.

As part of routine clinical management, various diagnostic tests were performed. These included blood sample analysis, complete blood count, peripheral blood smear, reticulocyte count, and specific tests such as malaria parasite (MP) smear, Typhi dot test, and Widal test, depending on the clinical suspicion. To further diagnose nutritional anemia, serum iron, serum ferritin, vitamin B12, and folic acid levels were measured. In cases where malignancy or bone marrow failure was suspected, bone marrow aspiration and biopsy were conducted. It is important to note that these investigations were performed as part of the standard diagnostic workup for pancytopenia and not as an intervention introduced by the study.

The study did not involve any interventions or experimental procedures beyond what was necessary for routine clinical care. The observational nature of the study was maintained, as the researchers solely recorded the findings from standard diagnostic and treatment procedures without altering or influencing the course of clinical management.

Statistical analysis

Data were entered into a master graphic using Microsoft Excel (Microsoft, Redmond, WA) and analyzed with SPSS version 20 (IBM SPSS, Armonk, NY). Descriptive statistics, including frequency analysis, percentage analysis, and mean analysis, were used to characterize categorical and continuous variables. Statistical information was presented as mean ± standard deviation (SD).

## Results

Table 1 summarizes the demographic and clinical characteristics of 70 study participants. The distribution by sex showed 30 (42.86%) male and 40 (57.14%) female participants. Age distribution ranged from nine (12.86%) in the one-month to one-year group, to 13 (18.57%) in those over 10 years old. Vaccination status indicated that 33 (47.14%) children had complete immunization, 22 (31.43%) had partial immunization, and 15 (21.43%) not vaccinated. The common symptoms included fever in 50 (71.43%), pallor in 42 (60%), joint pain in 39 (55.71%), and clinical signs such as hepatomegaly in 37 (52.86%) and splenomegaly in 35 (50%) were observed.

**Table 1:** Demographic and clinical characteristics of the study participants

Parameters	Category	Count	Percentage
Sex	Male	30	42.86%
	Female	40	57.14%
Age Range	1 month to 1 year	9	12.86%
	1-3 years	12	17.14%
	3-5 years	21	30%
	5-10 years	15	21.43%
	Over 10 years	13	18.57%
Vaccination Status	Complete	33	47.14%
	Partial	22	31.43%
	None	15	21.43%
Symptoms Presented	Fever	50	71.43%
	Pallor	42	60%
	Joint Pain (Arthralgia)	39	55.71%
	Growth Failure (Failure to Thrive)	13	18.57%
	Weight loss	11	15.71%
	Tremors	6	8.57%
	Jaundice	6	8.57%
	Vomiting	11	15.71%
Clinical Signs	Enlarged Liver (Hepatomegaly)	37	52.86%
	Enlarged Spleen (Splenomegaly)	35	50%
	Swollen Lymph Nodes (Lymphadenopathy)	12	17.14%
	Skin Darkening (Hyperpigmentation)	6	8.57%
	Bleeding	23	32.86%

Table 2 summarizes the prevalent hematological disorders in pediatric pancytopenia cases. Nutritional factors include iron deficiency anemia (five cases, 7.14%) and megaloblastic anemia (14 cases, 20%). Infectious causes include miliary tuberculosis (four cases, 5.71%) and systemic lupus erythematosus (five cases, 7.14%), which represent autoimmune disorders. Neoplastic conditions include acute lymphoblastic leukemia (six cases, 8.57%) and aplastic anemia (five cases, 7.14%), in addition to other causes such as hypoplastic bone marrow (two cases, 2.86%).

**Table 2:** A summary of hematological disorders and their prevalence in pediatric pancytopenia cases

Disorder Category	Diagnosis	Count	Percentage
Nutritional Deficiencies	Iron Deficiency	5	7.14%
	Vitamin B12 Deficiency Anemia	14	20%
	Combined Deficiency Anemia	3	4.29%
Infectious Diseases	Parvovirus Infection	1	1.43%
	Visceral Leishmaniasis	2	2.86%
	Human Immunodeficiency Virus (HIV)	1	1.43%
	Disseminated Tuberculosis	4	5.71%
	Typhoid Fever	1	1.43%
	Chronic Granulomatous Disorder	1	1.43%
	Severe Plasmodium Infection	1	1.43%
Autoimmune Conditions	Lupus Erythematosus	5	7.14%
	Idiopathic Thrombocytopenic Purpura	1	1.43%
	Autoimmune Hemolytic Anemia	3	4.29%
Neoplastic Disorders	Acute Lymphoblastic Leukemia (ALL)	6	8.57%
	Acute Myelogenous Leukemia (AML)	3	4.29%
	Aplastic Anemia	5	7.14%
Other Conditions	Bone Marrow Fibrosis	1	1.43%
	Bone Marrow Hypoplasia	2	2.86%
	Sideroblastic Disorder	1	1.43%

Table 3 presents the findings from bone marrow examinations conducted on 70 patients diagnosed with pancytopenia. Among them, 29 patients (41.43%) showed aplastic changes in their bone marrow, indicating decreased cell production across all lineages. Hyperplastic changes were observed in 20 patients (28.57%), characterized by increased cellular activity in response to blood cell deficiencies. Normal cellularity was found in 21 patients (30.00%), suggesting relatively intact bone marrow function despite the presence of pancytopenia.

**Table 3:** Bone marrow results in patients with pancytopenia

Bone Marrow Examination Result	Count	Percentage
Aplastic Changes	29	41.43%
Hyperplastic Changes	20	28.57%
Normal Cellularity	21	30%

## Discussion

Reduced levels of red blood cells, white blood cells, and platelets are the hallmarks of pancytopenia, a common clinical illness that is often seen in children. There are a number of underlying factors that can affect treatment and prognosis [6]. A thorough clinical history, physical examination, laboratory testing, and bone marrow examination are essential preconditions for determining its etiology [7], which is critical for early detection and treatment to lower mortality and morbidity.

The present study's results demonstrate that a decrease in all blood cell types can appear in various disorders among children, with a male-to-female ratio of 1:1.33. Similar studies reported ratios of 1:1.25 and noted male predominance in contrasting findings, possibly influenced by genetic, geographic, and nutritional factors [7,8]. Common presenting symptoms such as fever, pallor, arthralgias, and weight loss are consistent with literature reports [9-11], indicating disturbances in bone marrow cellularity that increase susceptibility to infections due to decreased cell counts and anemia-related symptoms.

Clinical features including hepatomegaly, splenomegaly, lymphadenopathy, and bleeding were frequently observed, consistent with reports from developing countries [11,12]. Megaloblastic anemia emerged as the predominant nutritional cause of pancytopenia, affecting 14 children (20%) in the study, often linked to malnutrition and deficiencies in vitamin B12 and folic acid [9]. Miliary tuberculosis was identified as the leading infectious cause, underscoring the importance of immunization and prompt antituberculosis treatment to prevent severe outcomes [10].

Infectious etiologies such as malaria, leishmaniasis, *Salmonella* infections, chronic granulomatous diseases, Parvovirus B19, and HIV were also noted, reflecting a broad spectrum of causes in pediatric pancytopenia [11-13]. Neoplastic causes, particularly acute lymphoblastic leukemia (ALL), were significant, consistent with findings from India and other developing countries reporting an incidence of 13-23% [9,11,14]. Less common causes included bone marrow failure, marrow fibrosis, and refractory anemia with ring sideroblasts.

Overall, the findings of the present study underscore the diverse etiological profile of pediatric pancytopenia and emphasize the importance of tailored preventive strategies and early interventions in clinical practice, aligning closely with previous research [15].

Limitations of the study

The single-center design of the current study is one of its limitations, which could limit how broadly the results can be applied. A multicenter strategy including several institutions could offer a more thorough knowledge of the prevalence of different pancytopenia etiologies. A thorough examination of the wider impacts on illness prevalence and presentation is further hampered by the study's shortcomings in not accounting for socioeconomic and cultural factors.

## Conclusions

The pediatric population exhibits a wide range of causes for pancytopenia, encompassing nutritional deficiencies, infectious diseases, autoimmune conditions, and malignancies. Among these, megaloblastic anemia emerges as the primary nutritional cause, while miliary tuberculosis stands out as the predominant infectious cause. Acute lymphoblastic leukemia (ALL) represents the most common malignant etiology of pancytopenia. The prevention strategies for infectious causes include enhancing vaccination coverage and administering broad-spectrum antibiotic treatment, which can aid in controlling infections. Dietary deficiencies can be corrected through nutritional enhancements and supplementation with cobalamin and iron. On the whole, although bone marrow failure and blood cancer are recognized as typical culprits of pancytopenia, this study highlights megaloblastic anemia as the leading cause. Early detection of these conditions is crucial for reducing morbidity and mortality among vulnerable pediatric patients. Recognizing these disorders as prevalent causes of pancytopenia ensures a timely initiation of appropriate diagnostic and therapeutic interventions, offering a more favorable prognosis than previously perceived.
